# Is time to plasma exchange the right question in suspected thrombotic thrombocytopenic purpura?

**DOI:** 10.1016/j.htct.2026.106478

**Published:** 2026-05-30

**Authors:** Rishi Raj Sinha

**Affiliations:** Department of Transfusion Medicine, CNC Blood Centre, Ground Floor, All India Institute of Medical Sciences (AIIMS), New Delhi, India

To the Editor,

I read with great interest the recent publication by Soares Ferreira Junior et al.: In patients with suspected thrombotic thrombocytopenic purpura, what is the optimal time to therapeutic plasma exchange? [1]

Therapeutic plasma exchange (TPE) remains the cornerstone of management in suspected thrombotic thrombocytopenic purpura (TTP), yet outcomes continue to vary widely despite a guideline-driven emphasis on early initiation [1] Recent population-level analyses using large administrative datasets have highlighted a non-linear relationship between time to TPE and outcomes, with only modest predictive capacity when timing is considered in isolation [1]. While these findings are important, interpreting temporal thresholds in isolation may overlook broader clinical and system-level determinants that influence outcomes in real-world practice.

From a transfusion medicine and apheresis perspective, suspected TTP represents a complex, multi-step care pathway, involving emergency physicians, haematologists, laboratories, blood banks, and apheresis services. Delays frequently arise not only before TPE initiation but also within the diagnostic-treatment interface, particularly related to diagnostic uncertainty, plasma availability, and coordination of apheresis services. These operational delays are rarely captured when time to TPE is defined solely by hospital admission or plasma issue time [2].

Importantly, clinical heterogeneity at presentation complicates the interpretation of time-based metrics. Disease severity, extent of neurologic or cardiac involvement, and comorbid organ dysfunction are well-recognised predictors of the outcome in TTP [3]. Patients with fulminant disease may experience poor outcomes despite early TPE, whereas others with less severe presentations may tolerate modest delays without adverse consequences. This heterogeneity may partly explain why timing alone demonstrates limited prognostic value in contemporary analyses [1].

Risk stratification tools such as the PLASMIC score provide an opportunity to refine early decision-making by identifying patients at highest likelihood of immune-mediated TTP when definitive ADAMTS13 testing is unavailable [4]. Incorporating rapid risk assessment into early workflows may be more clinically actionable than rigid adherence to time thresholds alone.

A complementary strategy is the implementation of a ‘TPE Readiness Bundle’ for suspected TTP, analogous to sepsis bundles. Such a model emphasises parallel processing rather than sequential delays and includes early haematology-transfusion medicine co-activation, standardised plasma allocation pathways, avoidance of prophylactic platelet transfusions, and predefined escalation for intensive monitoring [3,5]. Readiness-based strategies may reduce care variability even when the absolute time to TPE cannot be uniformly shortened (Table 1).

**Table 1:** Moving beyond ‘time to plasma exchange (TPE)’: a readiness-based framework for suspected thrombotic thrombocytopenic purpura (TTP) care.

Domain	Traditional time-based focus	Readiness-based approach (proposed)	Clinical value
Trigger for action	Fixed time threshold from admission	Early clinical suspicion + PLASMIC score	Reduces diagnostic inertia
Decision pathway	Sequential (diagnosis → plasma → TPE)	Parallel processing across teams	Minimises hidden delays
Risk stratification	Often implicit or delayed	Structured bedside risk scoring	Prioritises high-risk patients
Plasma availability	Reactive allocation	Pre-defined emergency plasma pathways	Prevents logistical delays
Transfusion practices	Variable platelet use	Standardised platelet avoidance protocols	Reduces iatrogenic risk
Apheresis activation	After confirmatory steps	Early co-activation with haematology	Improves workflow efficiency
Adjunctive therapy	TPE-centric	Integrated early immunotherapy/caplacizumab	Addresses disease biology
Quality indicators	Time to first TPE	Process metrics (consult time, readiness markers)	Enables audit and improvement
Applicability	High-resource settings	Adaptable across resource settings	Improves global relevance

Furthermore, the evolving therapeutic landscape necessitates reassessment of TPE-centric outcome models. The introduction of caplacizumab has altered early disease biology by rapidly inhibiting microvascular thrombosis and may modify the relationship between TPE timing and outcomes in selected patients [6]. Future studies should therefore evaluate interaction effects between TPE timing, adjunctive therapies, disease severity, and institutional readiness rather than evaluating time as an isolated predictor [1].

From a transfusion systems perspective, quality improvement efforts may benefit from shifting toward process-based indicators, such as time to haematology consultation, time to PLASMIC scoring, plasma availability latency, and appropriateness of component utilisation. These metrics are actionable, auditable, and adaptable across healthcare settings, including resource-limited environments [5].

In conclusion, while timely initiation of TPE remains essential, its impact should be interpreted within a broader, systems-based framework. A readiness-oriented approach that integrates clinical severity, diagnostic tools, transfusion infrastructure, and adjunctive therapies may offer greater potential to improve outcomes in suspected TTP rather than reliance on time thresholds alone.

## Interpretation

This framework emphasises that outcomes in suspected TTP are influenced not only by when TPE is initiated, but by how prepared the system is to deliver coordinated, risk-adapted care. A readiness-based approach offers actionable targets for quality improvement beyond rigid time threshol.

## Conflicts of interest

NIL.
